# Trans-septal delivery of hydrogel-encapsulated human umbilical cord MSC-derived neurospheres for acute neuroprotection in traumatic brain injury

**DOI:** 10.1063/5.0288289

**Published:** 2026-01-06

**Authors:** Dong Wook Kim, Ok Joo Lee, Bo Young Choi, Md. Tipu Sultan, Olatunji Ajiteru, Min Kyu Park, Ji Seung Lee, Soon Hee Kim, Kyu Young Choi, Sang Won Suh, Chan Hum Park

**Affiliations:** 1 Nano-Bio Regenerative Medical Institute, College of Medicine, Hallym University, 1 Hallymdaehak-gil, Chuncheon, Gangwon-do 24252, Republic of Korea; 2 Department of Physical Education, Hallym University, 1 Hallymdaehak-gil, Chuncheon, Gangwon-do 24252, Republic of Korea; 3 Institute of Sports Science, Hallym University, 1 Hallymdaehak-gil, Chuncheon, Gangwon-do 24252, Republic of Korea; 4 Department of Physiology, College of Medicine, Hallym University, 1 Hallymdaehak-gil, Chuncheon, Gangwon-do 24252, Republic of Korea; 5 Department of Otorhinolaryngology-Head and Neck Surgery, Hallym University College of Medicine, Kangnam Sacred Heart Hospital, Seoul 07441, Republic of Korea; 6 Department of Otorhinolaryngology-Head and Neck Surgery, Chuncheon Sacred Heart Hospital, School of Medicine, Hallym University, Chuncheon 24253, Republic of Korea

## Abstract

This study explores the therapeutic potential of hydrogel-encapsulated neurospheres derived from human umbilical cord mesenchymal stem cells (hUC-MSCs) in mitigating traumatic brain injury (TBI) and enhancing functional recovery in a rodent model. Trans-septal (intranasal) transplantation of these neurospheres demonstrated significant neurological improvement, reduced neuronal damage, and preserved neuronal structures and functions. The hUC-MSCs cultured in a customized bioreactor retained essential MSC characteristics, including marker expression and multi-lineage differentiation potential, ensuring their therapeutic efficacy. Following neural induction, hUC-MSCs formed neurospheres that promoted cell aggregation, differentiation, and neuroprotective effects. Encapsulation within a hydrogel provided a stable environment, significantly reducing TBI-induced cell death in co-cultured HT22 cells and improving *in vivo* outcomes. Therapeutic benefits were evidenced by decreased modified neurological severity scores (mNSS), improved ΔmNSS, and restoration of neurotrophic factors, such as brain-derived neurotrophic factor in the hippocampal CA1 region, which supports cognitive and memory functions. Immunohistochemical and immunofluorescence analyses confirmed the neurospheres' ability to mitigate TBI-induced neurodegeneration, oxidative stress, and dendritic damage. Reduced neurodegenerative markers and preserved dendritic integrity, particularly microtubule-associated protein 2 expression in the CA1 and dentate gyrus (DG) regions, underscore the potential of hUC-MSC–derived neurospheres in maintaining neural connectivity and function after TBI.

## INTRODUCTION

The human central nervous system (CNS) is highly susceptible to injury, often resulting in severe and long-lasting neurological deficits.^1^ Traumatic brain injury (TBI) can result in a variety of impairments, such as difficulties with movement, thinking, and emotional regulation.^2^ Even mild TBI can have significant long-term consequences, such as hippocampal changes and cognitive impairment.^3^ While various treatment strategies exist, their efficacy in promoting neural repair and functional recovery is often limited. Secondary injury processes, which occur in the days and weeks following the initial injury, further exacerbate neuronal damage and contribute to persistent neurological deficits.^4–6^

Apoptosis, or programmed cell death, is a vital process for normal development and tissue maintenance. However, when dysregulated, excessive apoptosis contributes to various neurodegenerative diseases, such as Parkinson's, Alzheimer's, and Huntington's diseases.^7–9^ Neurogenesis, once thought limited to development, persists in adults, especially in the hippocampus. It is crucial for learning, memory, and mood regulation and can be regulated after brain injuries. Understanding apoptosis and neurogenesis may lead to neuronal survival and regeneration therapies in neurological disorders.^10–12^ Brain damage stems from cellular dysfunction and apoptosis, leading to cognitive and emotional problems. Therefore, TBI research focuses on finding effective treatments, of which current clinical studies aim to lessen these impairments and improve cognitive and motor recovery.^13^ Despite advances in neuroprotective strategies and acute clinical management, clinical translation remains limited by treatment-related adverse effects and the overall complexity of the injury. Current therapeutic approaches largely focus on preserving at-risk neurons, which may be insufficient in cases of extensive neuronal loss. In such settings, regenerative strategies that promote neuronal replacement or support exogenous cell transplantation may be required to achieve meaningful recovery.

Genetically engineered neural stem/progenitor cells, including those derived from embryonic or adult stem cells, have shown promise in improving neurological outcomes in preclinical models of brain injury.^14^ Likewise, neural stem cell (NSC) transplantation enhances motor recovery after TBI in rodent models.^15^ Furthermore, bone marrow stromal cell transplantation reduces functional deficits and apoptosis while promoting angiogenesis, synaptogenesis, and neurogenesis in stroke rodent models.^16,17^ Neural stem cell transplantation in rodents promotes survival, integration, and differentiation, replacing lost cells and improving TBI outcomes, highlighting stem cell therapy's potential for neurological disorders.^18–20^

While the human CNS has limited regenerative capacity, there has been growing interest in stem cell-based therapies to treat neurological disorders, including TBI.^21,22^ Neural stem cells, derived from embryonic or adult tissue, have shown promise in preclinical studies.^23,24^ When transplanted into injured brains, these cells can differentiate into neurons and glia, potentially replacing lost cells and restoring function.^25^ However, the limited availability of neural stem cells and ethical concerns associated with their use have spurred research into alternative cell sources.^26,27^ Mesenchymal stem cells (MSCs), derived from various tissues, such as bone marrow, umbilical cord blood, and adipose tissue, have emerged as a promising cell source for cell therapy.^28–30^ MSCs can be easily isolated, expanded in culture, and transplanted without significant immune rejection. Moreover, they possess the ability to differentiate into neural cells under specific conditions.^31,32^ While MSC-based therapies have shown some success in preclinical studies, their efficacy in promoting neural regeneration and functional recovery in TBI patients remains to be fully established.^29^ Further research is needed to optimize cell differentiation protocols, improve cell survival and integration, and develop effective delivery strategies to maximize the therapeutic potential of stem cell-based therapies for TBI.

Stem cell-based therapies face several challenges, including low cell survival rates and limited differentiation into specific neuronal subtypes at injury sites. While stimulating endogenous neural stem cells can aid recovery, it may be insufficient for full restoration. Effective stem cell delivery is crucial, and hydrogels have emerged as promising biomaterials for neural tissue engineering.^33^ Hydrogels support cell growth, migration, and differentiation. Encapsulating stem cells in hydrogels enhances their survival, enables targeted delivery, and regulates the release of growth factors.^33^

Traumatic brain injury (TBI) often involves extensive neuronal loss, while current cell therapies face critical barriers, including poor cell survival, inefficient delivery, and low retention at the injured brain. To address these gaps, we developed a scalable workflow that generates human umbilical cord mesenchymal stem cell (hUC-MSC)-derived neurospheres and encapsulated them in a silk fibroin/GelGMA hydrogel for trans-septal (intranasal) administration.^33^ Building on evidence that MSC neurospheres exhibit enhanced neurogenic differentiation potential and migratory capacity compared with dissociated single cells,^34–36^ we hypothesized that pre-formed neurospheres embedded in a brain-compatible hydrogel (hUC-MSC derived neurospheres, HMNS) would enhance acute outcomes by improving cell viability, local retention, and paracrine support—thereby reducing neuronal degeneration and oxidative/nitrosative stress and preserving dendritic integrity in the injured hippocampus. To avoid the invasiveness of direct brain administration, trans-septal (intranasal) administration was used to deliver the stem cell-laden hydrogel.

## RESULTS

### Phenotype and cellular characterization of hUC-MSCs

**FIG. 1.**
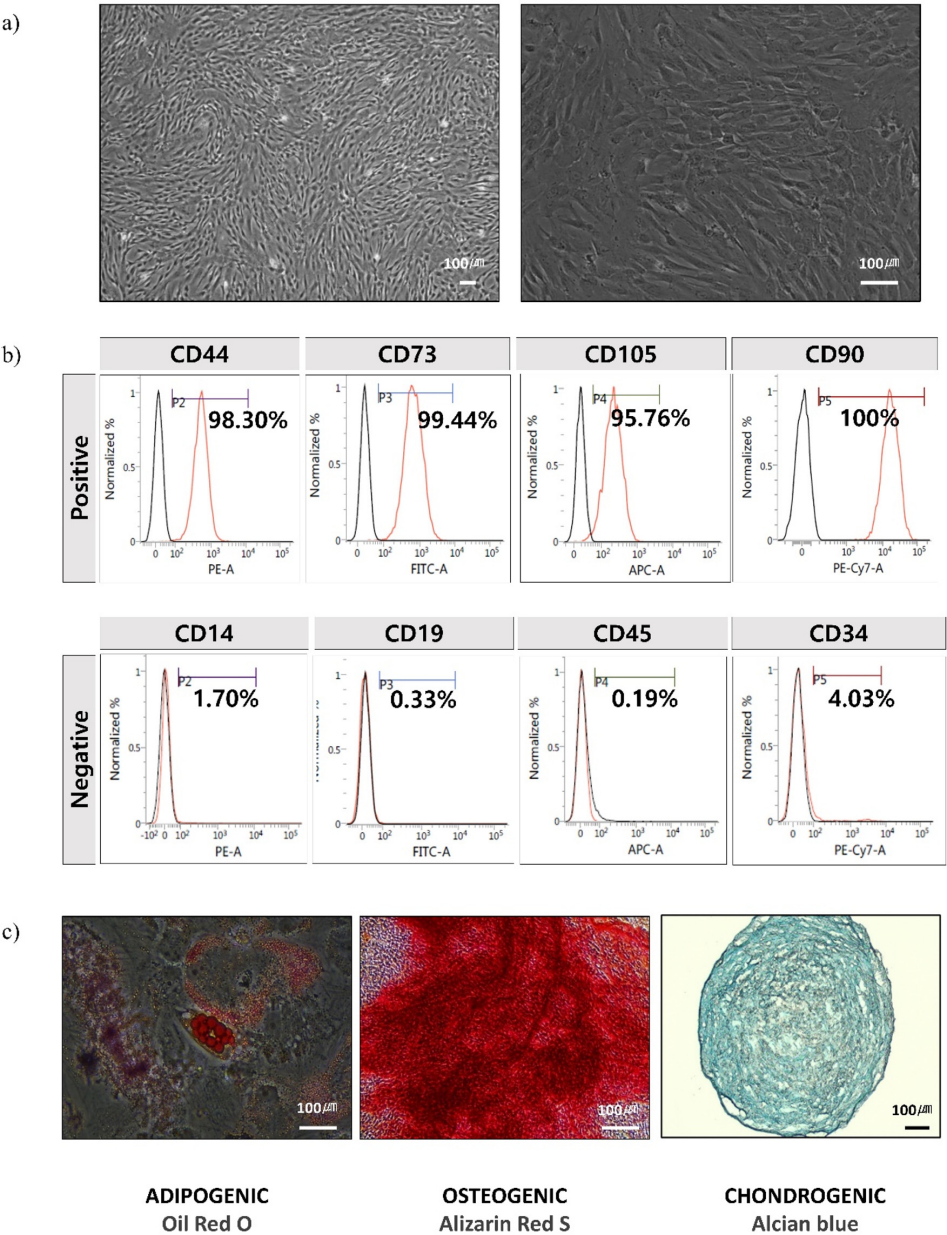
Characterization of human umbilical cord mesenchymal stem cells (hUC-MSCs). The hUC-MSCs characterization was investigated by morphology, flow cytometry analysis, and differentiation. (a) Microscopic images of hUC-MSCs cultured in a bioreactor. (b) Flow cytometry analysis of cell surface markers present on hUC-MSCs cultured in a bioreactor. Bioreactor-cultured hUC-MSCs were found to be positive for the hUC-MSCs markers CD44, CD73, CD90, and CD105 and negative for the markers CD14, CD19, CD34, and CD45. (c) Adipocyte differentiation was confirmed by Oil Red O staining, osteoblast differentiation by alizarin red S staining, and chondrocyte differentiation by Alcian Blue staining. Scale bar: 100 μm.

Cells that are isolated, expanded in a bioreactor, and subsequently cryopreserved typically exhibit a spindle-shaped morphology characteristic of MSCs [Fig. 1(a)]. The phenotyping of bioreactor culture MSCs was performed using flow cytometry (FACSVerse; BD Biosciences, San Jose, CA, USA). Flow cytometry analysis confirmed the expression of specific mesenchymal stem cell surface markers, including primitive cell markers, such as CD44, CD73, and CD90, while being negative for hematopoietic markers like CD14, CD19, CD34, and CD45 [Fig. 1(b)]. These results indicate that bioreactor culture did not alter the surface phenotype of MSCs. The multi-lineage differentiation potential of bioreactor-cultured MSCs was demonstrated by their ability to differentiate into adipocytes, osteoblasts, and chondrocytes, as confirmed by Oil Red O, Alizarin Red S, and Alcian Blue staining, respectively [Fig. 1(c)].

### Neurosphere induction of hUC-MSCs

**FIG. 2.**
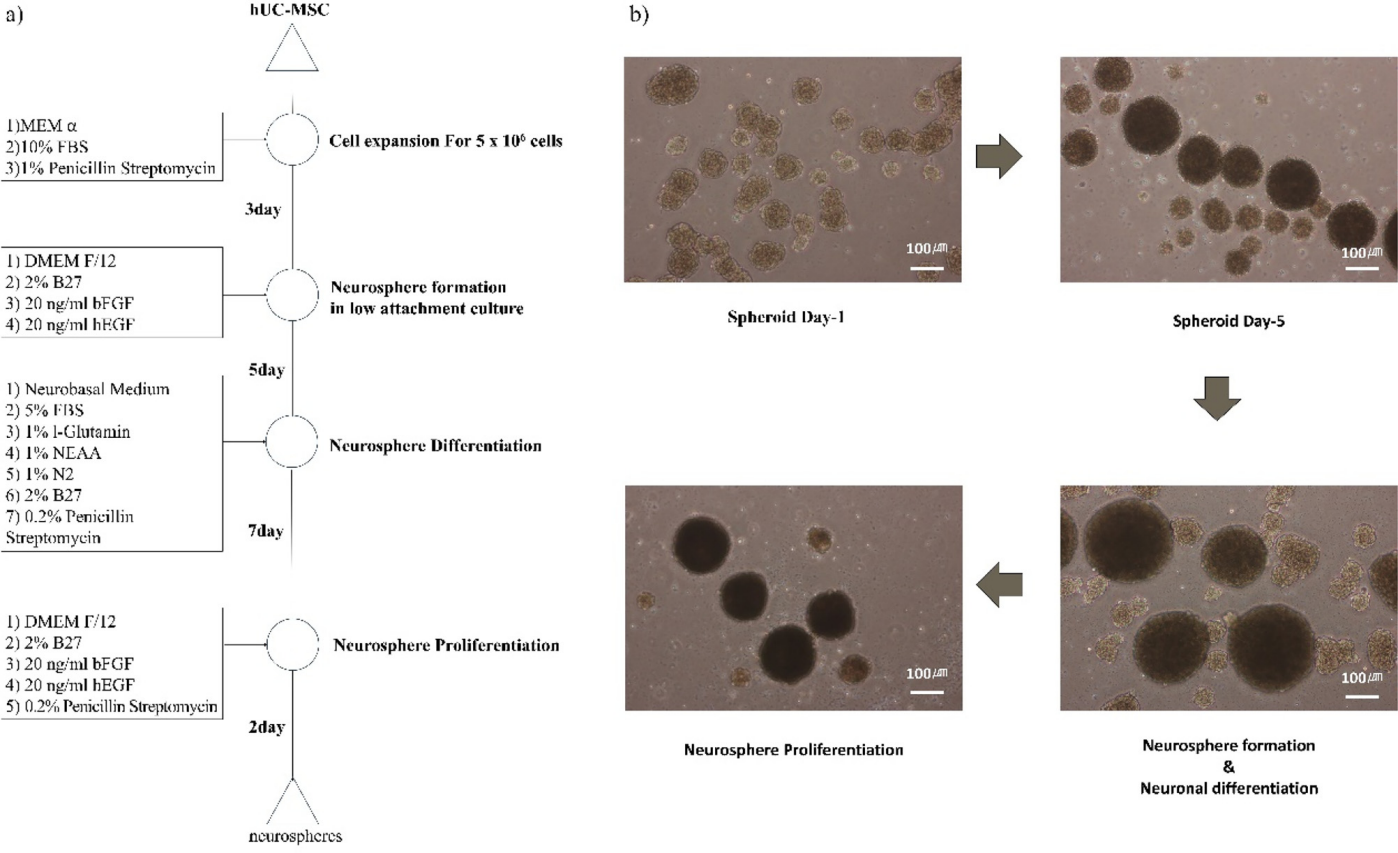
Generation of neurospheres from hUC-MSCs. (a) Schematic workflow for differentiating human umbilical cord mesenchymal stem cells (hUC-MSCs) into 3D neurospheres, showing key culture stages' timeline and medium conditions. (b) Temporal progression of neurosphere formation, demonstrating the spheroid generation and differentiation of hUC-MSCs spheroids into neural progenitor aggregates and subsequent neuronal proliferation. Scale bar: 100 *μ*m.

The stepwise protocol for generating neurospheres from thawed 2D-cultured hUC-MSCs is illustrated in Fig. 2(a), detailing the timeline and culture conditions required for each phase of spheroid formation and proliferation. Following thawing, hUC-MSCs were expanded as monolayers and harvested within 5–8 passages. The cells were then cultured in spheroid formation medium within ultra-low attachment plates, where they began aggregating within 24 h, with robust spheroid formation observed by day 5 [Fig. 2(b)]. To induce neuronal differentiation, spheroids were transferred to neurodifferentiation medium containing 5% fetal bovine serum (FBS), 1% L-glutamine, 1% non-essential amino acids (NEAA), 1% N2 supplement, 2% B27, and 0.2% penicillin/streptomycin for 7 days. Subsequent return to spheroid formation medium resulted in further maturation, yielding larger differentiated neurospheres with enhanced structural integrity [Fig. 2(b)].

Previous studies have reported that MSC-derived neurospheres (including those from umbilical cord) can upregulate neural stem/progenitor–associated markers (e.g., SOX2, Nestin) and exhibit enhanced neurogenic potential compared with dissociated MSCs.^34–36^ In the present work, we, therefore, refer to our constructs as hUC-MSC–derived neurospheres (HMNS). However, we did not directly assess these markers in our preparations and do not infer a formal lineage conversion.

### HMNS attenuate injury in the *in vitro* TBI-mimicking scratch model

To evaluate the regenerative potential of neurospheres, two complementary *in vitro* assays were performed using distinct cell lines selected to best match each experimental end point. HT22 cells, derived from the hippocampus, were used for viability assays as a relevant model for TBI-induced neuronal injury, whereas Neuro-2a cells, characterized by high motility, were used for migration assays to quantify wound closure dynamics.

**FIG. 3.**
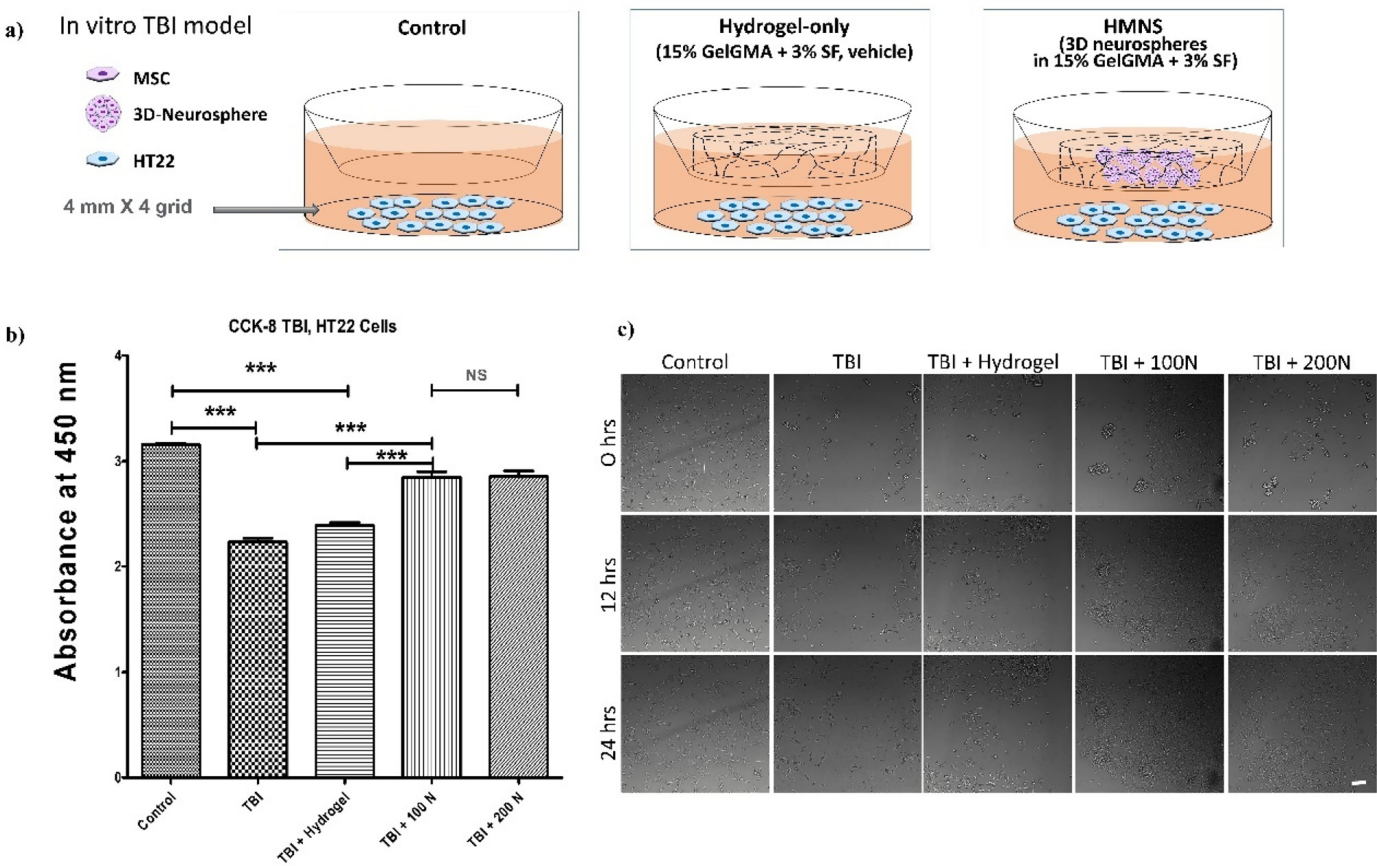
HMNS attenuate TBI-induced HT22 cell death *in vitro*. (a) Schematic illustration of the *in vitro* co-culture system used to evaluate neuroprotective effects. HT22 cells subjected to a scratch injury were co-cultured with transwell inserts containing either an acellular silk-fibroin/GelGMA hydrogel (hydrogel-only vehicle, no MSCs) or pre-formed hUC-MSC–derived neurospheres encapsulated within the same hydrogel (HMNS therapeutic group). (b) Cell viability assessed by Cell Counting Kit-8 (CCK-8) assay after 48 h. Data are presented as mean ± SEM (n = 3 independent cultures per group). Statistical significance was determined by one-way analysis of variance (ANOVA) followed by Tukey's post hoc test. p < 0.001 vs the TBI + hydrogel-only group. The difference between the 100 and 200 N groups was not significant (ns). (c) Representative images of Neuro2a cell migration in a scratch assay at 0, 12, and 24 h, comparing: (i) uninjured control, (ii) scratch + hydrogel-only (15% GelGMA + 3% SF), and (iii) scratch + hydrogel-encapsulated neurospheres (100 N or 200 N). Scale bar: 100 *μ*m.

Mechanical scratch injury (to mimic TBI) significantly reduced HT22 neuronal cell viability, while treatment with neurosphere-encapsulated hydrogel (100 and 200 N) significantly enhanced cell viability compared to the hydrogel-only control. Although no statistically significant difference was found between the 100 and 200 N groups, the 200 N condition exhibited a modest but consistent trend toward enhanced cell viability. Therefore, 200 N was selected for subsequent *in vivo* experiments as the representative therapeutic dose [Fig. 3(b)].

A wound-healing assay was then performed using Neuro-2a cells co-cultured under the following conditions: control (no scratch), 15% GelGMA + 3% SF hydrogel only, and HMNS (100 N or 200 N) encapsulated within the same hydrogel [Fig. 3(a)]. These co-cultures were maintained for 0, 12, and 24 h, and images were acquired using an Incell Analyzer System. Neurosphere-encapsulated hydrogels promoted cell migration, with the 200 N condition exhibiting the most pronounced wound closure at 24 h [Fig. 3(c)].

### Trans-septal transplantation of hydrogel-encapsulated HMNS exerts neuroprotective effects and improves neurological function after TBI

**FIG. 4.**
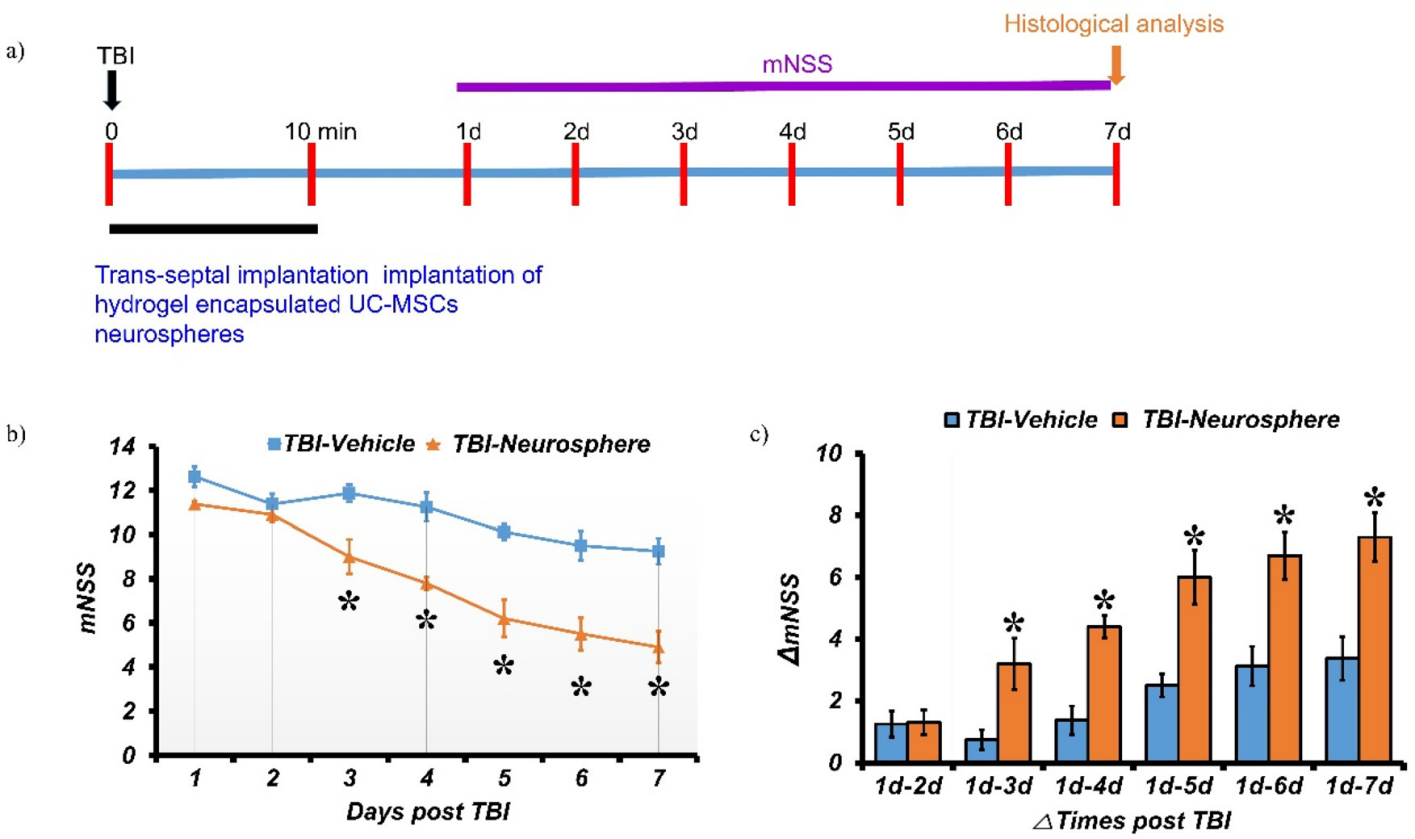
Trans-septal implantation of hydrogel-encapsulated HMNS reverses TBI-induced neurological impairment in rat model of TBI. (a) Schematic timeline of the experimental design for the *in vivo* TBI. (b) Behavioral neurological impairment was assessed via mNSS and (c) evaluated by measuring the change in mNSS (ΔmNSS) at various time points between day 1 and day 7 post-injury. Data are presented as mean ± SEM (n = 6 animals per group). Statistical significance was determined by two-way repeated measures ANOVA followed by post hoc comparisons. *p* < 0.05 vs the hydrogel-only group.

The therapeutic efficacy of trans-septal transplantation of HMNS encapsulated in hydrogels as a therapeutic strategy for enhancing functional recovery and minimizing neurological damage after TBI was evaluated using a rodent model in a well-defined timeline for behavioral and histological analyses [Fig. 4(a)]. The modified Neurological Severity Score (mNSS) was used to evaluate neurological outcomes. To analyze the time course of neurological recovery, the changes in mNSS were tracked and ΔmNSS was calculated [Figs. 4(b) and 4(c)]. When neurological function was evaluated at specific time points after traumatic brain injury (TBI), the experimental group transplanted with hUC-MSC neurospheres encapsulated in hydrogels showed a statistically significant decrease in mNSS scores [Fig. 4(b)] and an increase in ΔmNSS scores [Fig. 4(c)], indicating a considerable improvement in neurological recovery compared to the control group. After completing the behavioral tests, the experimental animals were sacrificed, and their brain tissues were extracted and cryosectioned. An immunohistochemical analysis was performed using brain-derived neurotrophic factor (BDNF) and NeuN antibodies. In addition, we investigated whether trans-septally delivered hUC-MSC neurospheres induce increased expression of BDNF and neuroprotective effects in the bilateral hippocampus 7 days after TBI.

**FIG. 5.**
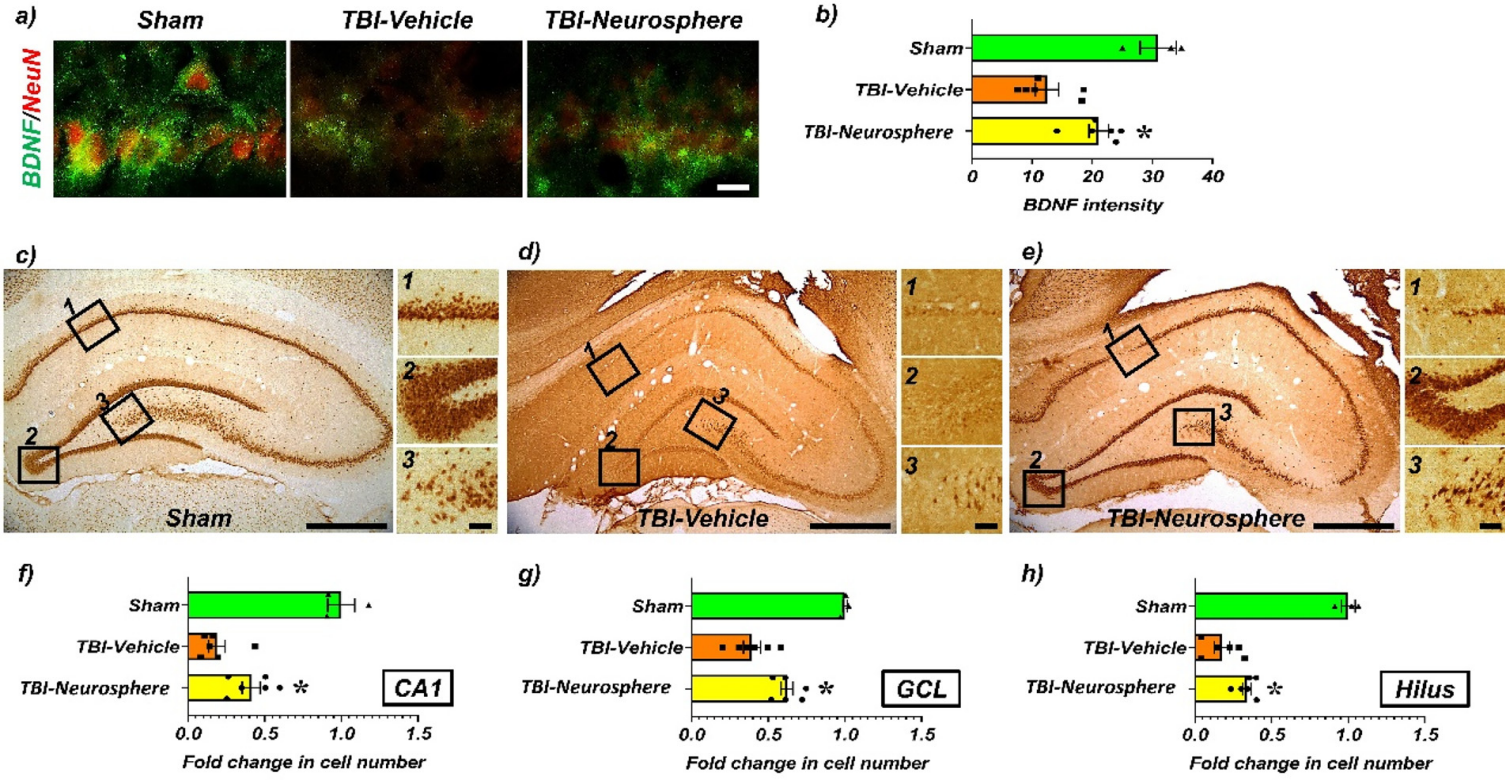
Neuroprotective effects of trans-septal transplantation of HMNS following TBI. (a) Representative images show tissue samples stained for BDNF and NeuN markers 7 days after TBI. Scale bar, 20 μm. (b) Quantification of BDNF intensity in the CA1 region of the ipsilateral hippocampus stained for mature BDNF. (c)–(e) Immunostaining with NeuN antibody revealed the distribution of live neurons in the ipsilateral hippocampal CA1 region. Scale bar, 50 μm. (f)–(j) Quantification of the number of NeuN+ neurons from each hippocampal area. Data for all quantifications are presented as mean ± SEM (n = 3 for sham, n = 6 for TBI groups). Statistical significance was determined by one-way ANOVA followed by Tukey's post hoc test. *p < 0.05 vs the hydrogel-only group.

Compared to the control group (sham group), the TBI-induced experimental group exhibited a significant decrease in BDNF expression in the hippocampal CA1 region. However, in the TBI experimental group that received HMNS encapsulated in hydrogel, there was a notable recovery in BDNF expression [Figs. 5(a) and 5(b)]. Additionally, a significant increase in NeuN-positive neurons was observed in the CA1, dentate gyrus granule cell layer (GCL), and hilus regions of the hippocampus [Figs. 5(c) and 5(h)]. These findings suggest a marked improvement in neuronal survival rates.

### Trans-septal transplantation of HMNS reduces nitrosative stress, oxidative stress, and dendritic cell loss after TBI

Next, we performed immunofluorescence staining using Fluoro-Jade B (FJB), nitrotyrosine, 4-hydroxynonenal (4-HNE), and microtubule-associated protein 2 (MAP2) antibodies to detect neuronal death, nitrosative stress, oxidative stress, and dendritic damage following TBI. Brain tissue samples were collected 7 days post-TBI.

**FIG. 6.**
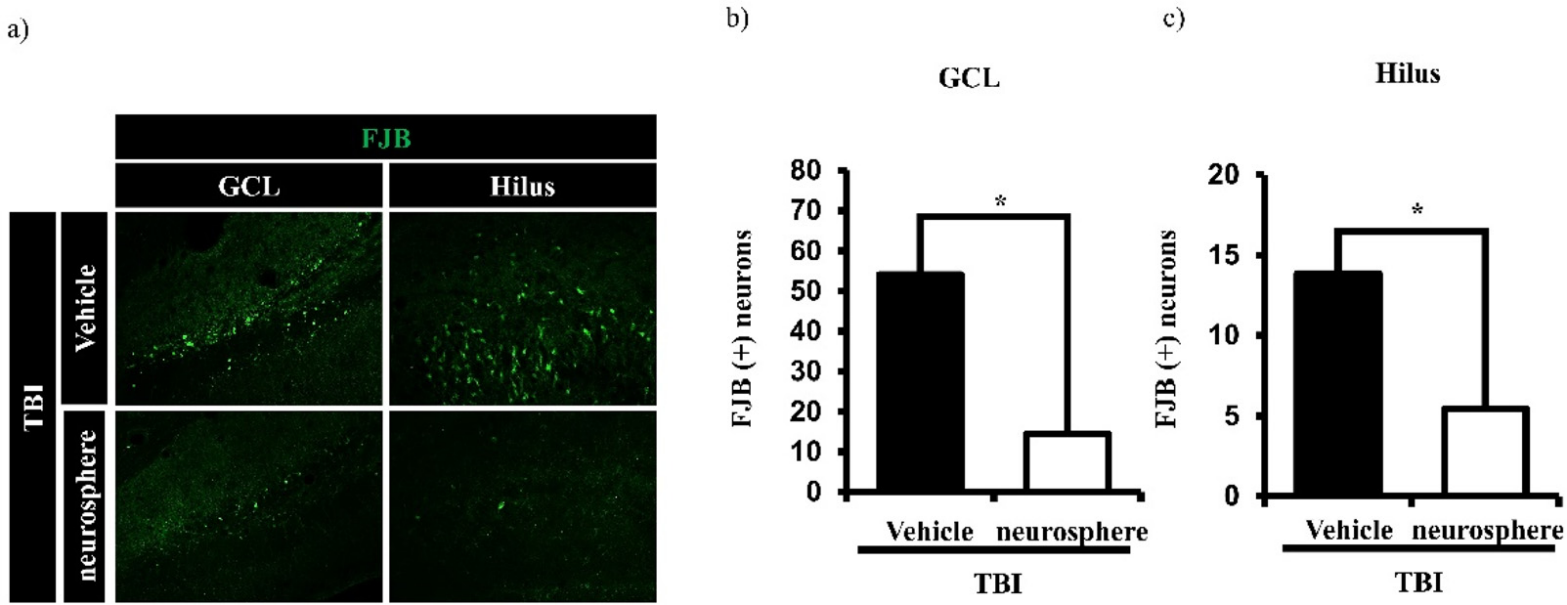
Trans-septal transplantation of HMNS reduces acute neuronal death after TBI. (a) Representative images of FJB staining in the hippocampal GCL, and hilus regions. (b) Quantification of FJB+ neurons in the hippocampal GCL, and (c) hilus regions. Data are presented as mean ± SEM (n = 6 animals per group). Statistical significance was determined by an unpaired t-test. p < 0.05 vs the hydrogel-only group. Scale bar: 100 μm.

The neuroprotective effect of hydrogel-encapsulated HMNS transplantation was investigated by analyzing neuronal cell death in the ipsilateral granule cell layer (GCL) and hilus of the hippocampus after traumatic brain injury (TBI). Degenerated neurons were identified using Fluoro-Jade B (FJB) staining [Fig. 6(a)]. The TBI-only group showed a significant increase in FJB-positive neurons in both the GCL and hilus compared with the sham group. However, the HMNS transplantation group showed a significant decrease in FJB-positive neurons compared to the hydrogel-only group [Figs. 6(b) and 6(c)].

**FIG. 7.**
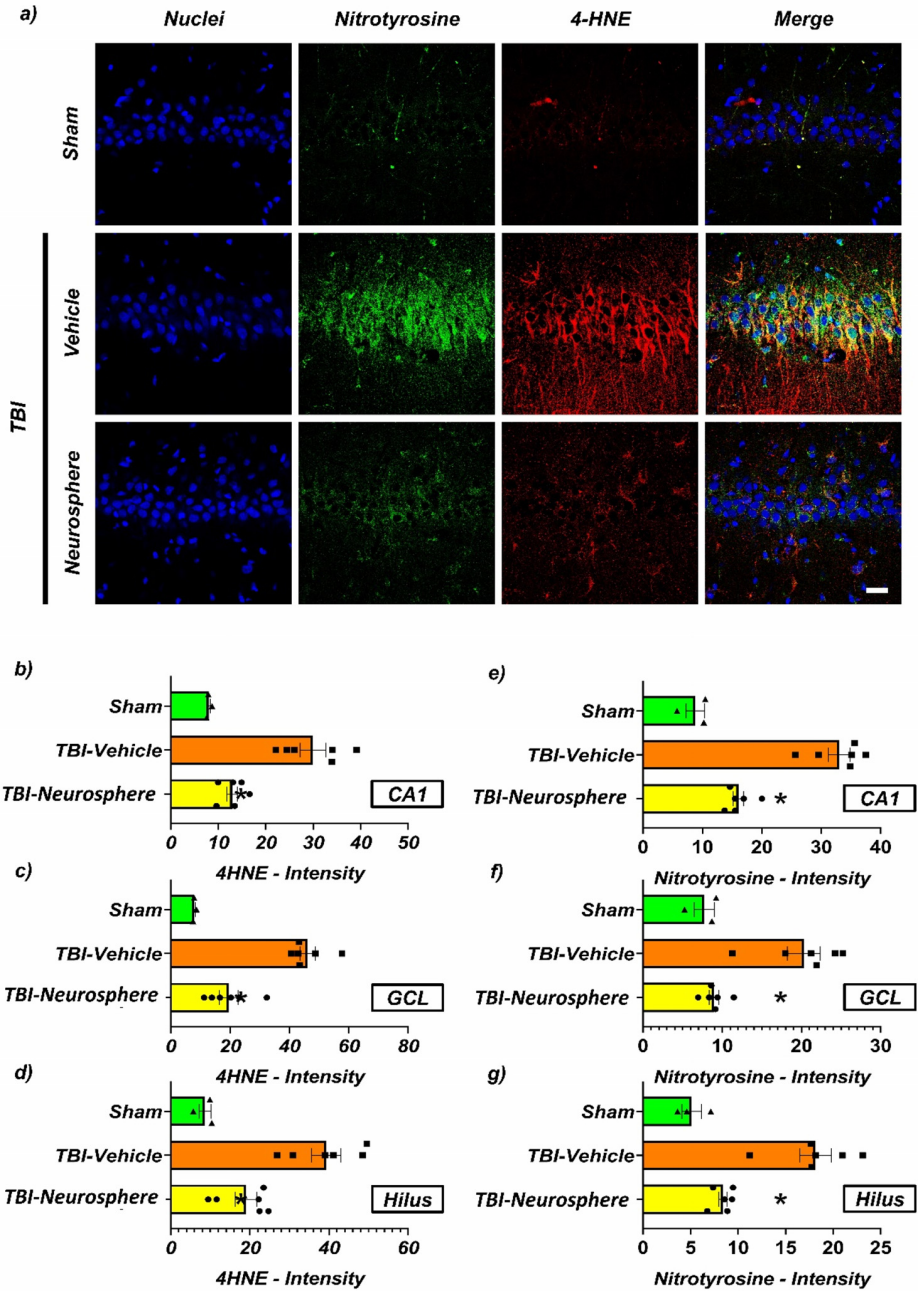
Neurosphere therapy attenuates oxidative stress in the hippocampus post-TBI. (a) Representative images demonstrate the distribution of oxidative stress markers across experimental groups, with sections stained for nuclear DNA (DAPI, blue), protein nitrosylation (nitrotyrosine, green), and lipid peroxidation (4-HNE, red). The merged channels illustrate the spatial relationship between these markers in Sham (uninjured), TBI-Vehicle (hydrogel-treated), and TBI-Neurosphere (HMNS-treated) groups. Qualitative assessment shows elevated nitrotyrosine and 4-HNE signals in TBI-Vehicle animals compared to Sham controls, with notable reduction in the neurosphere-treated group. Quantitative analysis of 4-HNE fluorescence intensity demonstrated significant oxidative damage in all hippocampal subregions following TBI. The (b) CA1 pyramidal layer, (c) dentate gyrus granule cell layer (GCL), and (d) hilus all showed marked increases in 4-HNE signal in vehicle-treated TBI animals compared to Sham controls (p < 0.05). Neurosphere treatment significantly attenuated these increases, particularly in the GCL and hilus (p < 0.05 vs TBI-Vehicle). Nitrotyrosine intensity measurements revealed TBI-induced protein nitrosative damage across the same subregions: CA1 (e), GCL (f), and hilus (g). While vehicle-treated TBI animals showed robust increases vs Sham (p < 0.01), neurosphere administration substantially mitigated these effects, with the most pronounced protection observed in the CA1 and GCL regions (p < 0.05 vs TBI-Vehicle). Data are presented as mean ± SEM (n = 3–4 animals per group). Statistical significance was determined by one-way ANOVA followed by Tukey's post hoc test. p < 0.05 vs TBI-Vehicle; #p < 0.05 vs Sham.

The expression of nitrotyrosine and 4-hydroxynonenal (4-HNE), which are oxidative and nitrosative stress markers, in the ipsilateral hippocampus of rats induced by traumatic brain injury (TBI), was analyzed by immunofluorescence staining. The experimental group transplanted with hUC-MSC neurospheres showed a notable decrease in nitrotyrosine and 4-HNE levels compared to the control group that was administered only hydrogel [Fig. 7(a)]. Quantitative analyses results showed that the hUC-MSC neurosphere transplant group showed significantly lower staining intensities of nitrotyrosine and 4-HNE [Figs. 7(b) and 7(g)].

**FIG. 8.**
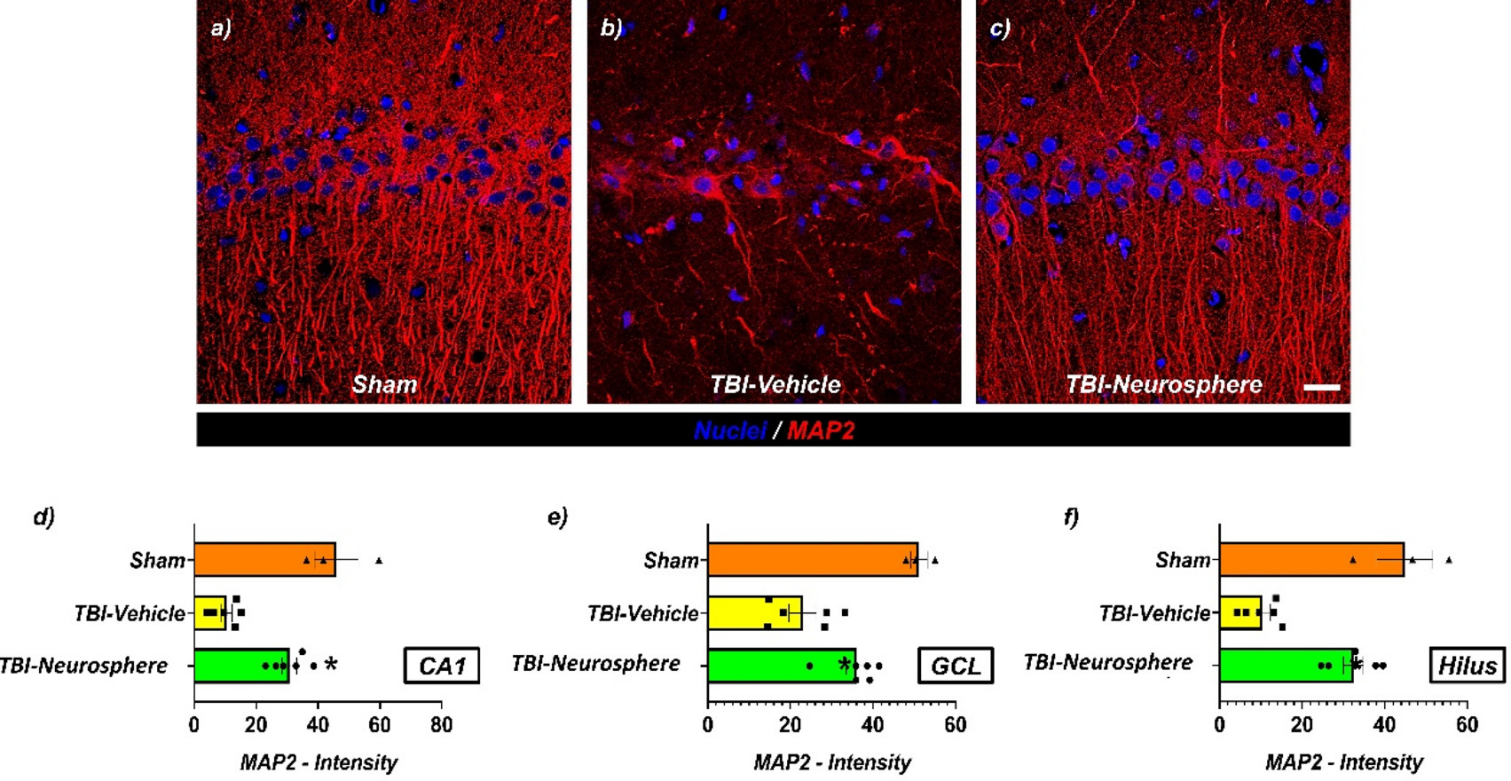
Neurosphere treatment enhances MAP2 expression in the hippocampus following traumatic brain injury (TBI). (a)–(c) Representative immunofluorescence images of hippocampal sections stained for MAP2 (microtubule-associated protein 2, red) and nuclei (DAPI, blue) in the Sham (a), TBI-Vehicle (b), and TBI-Neurosphere (c) groups. Scale bar: 20 μm. (d)–(f) Quantification of MAP2 fluorescence intensity in hippocampal subregions: (d) CA1, (e) granule cell layer (GCL), and (f) hilus. Data are presented as mean ± SEM (n = 3 for sham, n = 6 for TBI groups). Statistical significance was determined by one-way ANOVA followed by Tukey's post hoc test. **p < 0.05, ***p < 0.01 vs Sham.

The degree of dendritic damage after TBI was confirmed by immunostaining with microtubule-associated protein 2 (MAP2) antibody. The TBI-only group showed a decrease in MAP2 expression in the CA1 and dentate gyrus (DG) regions of the hippocampus. It is noteworthy that hUC-MSC neurospheres encapsulated in hydrogels effectively alleviated this dendritic loss [Figs. 8(a) and 8(f)].

## DISCUSSION

Traumatic brain injury (TBI) triggers an immune response that releases inflammatory molecules, creating a hostile microenvironment that hinders tissue repair and regeneration.^4^ The effects of brain injury can persist for months or even years after the initial trauma, potentially leading to irreversible neuronal loss, even in mild to severe cases.^5^ Persistent inflammation and ongoing neuronal degeneration following TBI are associated with an increased risk of developing neurodegenerative diseases.^6^ Additionally, TBI significantly impairs motor function, cognitive abilities, and emotional regulation.^5^ However, current treatments offer limited efficacy in promoting neural regeneration and functional recovery. Despite advancements in clinical management, no pharmacological treatments are currently available for TBI.^13^

A crucial aspect of developing neuroprotective and regenerative therapies involves regulating excessive cell death and promoting neurogenesis. Given the irreversible nature of many neurological disorders and the central nervous system's limited regenerative capacity, stem cell-based therapies have garnered significant attention in recent decades as a promising strategy for treating neurological injuries, including TBI.^37^

Notably, mesenchymal stem cells (MSCs) offer a novel therapeutic approach for TBI due to their immunomodulatory and neuroprotective properties. MSCs mitigate excessive inflammatory responses post-TBI by suppressing the production of pro-inflammatory cytokines while promoting the release of anti-inflammatory cytokines, thereby improving the microenvironment of damaged brain tissue to facilitate neuroregeneration.^38^ Furthermore, MSCs secrete neurotrophic factors that enhance the survival of injured neurons, inhibit neuronal apoptosis, and stimulate the differentiation of neural stem cells, contributing to the regeneration of damaged neural tissue.^30^

MSCs can be administered through various routes, including intravenous injection, intraventricular infusion, and direct injection into the lesion site, allowing for personalized treatment tailored to the patient's condition. Among the various routes, transnasal delivery can offer a feasible, minimally invasive method for delivering agents, including MSCs, for the treatment of TBI.^33^ Recent research indicates that encapsulating MSCs within hydrogels for targeted delivery enhances cell viability and enables precise administration, maximizing therapeutic efficacy while minimizing adverse effects. Additionally, culturing umbilical cord-derived MSCs (UC-MSCs) as neurospheres enhances their neurogenic differentiation and migratory capacity, further promoting cell migration and regeneration within damaged brain tissue.^34^

Therefore, this study aims to evaluate the therapeutic efficacy of transplanting hydrogel-encapsulated HMNS for TBI. To achieve this, neurospheres generated from large-scale cultured MSCs will be encapsulated in SF and GelGMA-based hydrogels and transplanted into the nose for trans-septal delivery.

Bioreactor-cultured hUC-MSCs met the criteria for MSCs: they exhibited a spindle-shaped morphology, were positive for MSC markers (CD44, CD73, CD90, and CD105) and negative for hematopoietic markers, and demonstrated trilineage differentiation potential (adipocytes, osteoblasts, and chondrocytes).

We successfully induced human umbilical cord-derived mesenchymal stem cells (hUC-MSCs) to form neurospheres. Initially, the hUC-MSCs presented as a monolayer of large, flat cells. Upon culturing in spheroid formation medium on ultra-low attachment plates, cell aggregation was observed on day 1, with significant spheroid formation by day 5. Following neurodifferentiation, the neurospheres were returned to the spheroid formation medium to facilitate growth. The neurosphere induction process was conducted based on our previous research, and the data are not presented in this report.^34–36^

To enhance cell viability, structural stability post-transplantation, and overall therapeutic efficacy, the HMNS were encapsulated within a composite hydrogel containing 3% (w/v) SF and 15% (w/v) GelGMA. Hydrogel encapsulations are known to provide a more stable environment for cells compared to cells alone, thereby improving cell viability and therapeutic outcomes. Based on an internal pilot titration (unpublished), we selected two inocula—100 and 200 neurospheres—for subsequent experiments per sample treatment. *In vitro*, TBI-induced injury significantly reduced HT22 cell viability. However, treatment with the neurosphere-encapsulated hydrogel (both 100 and 200 N) significantly enhanced cell viability. While a non-significant trend favored the 200 N group, the difference between the two doses was not statistically significant. Furthermore, a wound healing assay using Neuro2a cells co-cultured with neurosphere-encapsulated hydrogels (100 and 200 N) revealed that the neurospheres significantly promoted cell migration, with the 200 N condition exhibiting the most significant wound closure at 24 h. Prior literature indicates that HMNS display NSC-associated features at marker and behavioral levels.^34–36^ Our findings are compatible with these features; nevertheless, we interpret them conservatively and avoid inferring definitive NSC identity.

To evaluate the therapeutic efficacy of trans-septal delivery of HMNS in a rodent model of traumatic brain injury (TBI), neurological outcomes were assessed using the modified neurological severity score (mNSS). HMNS-treated animals showed significant functional recovery compared with controls, indicating that HMNS transplantation enhances neurological repair after TBI. These results are consistent with the previous findings that MSC-based therapies promote neural regeneration and functional improvement through anti-inflammatory and anti-apoptotic mechanisms.^39^

Immunohistochemical analyses demonstrate that TBI significantly reduced BDNF expression in the CA1 region of the hippocampus, as observed in the TBI-only group.^40^ BDNF is a key neurotrophic factor involved in neuronal growth, survival, and synaptic plasticity, making it essential for brain repair processes following injury.^41^ The group that received HMNS transplants, however, exhibited a marked restoration of BDNF expression in the CA1 region, suggesting that these transplanted cells might create an environment conducive to endogenous neurotrophic support. Additionally, the increased number of NeuN-positive neurons in regions, including the CA1, GCL, and hilus, further supports the neuroprotective effect of HMNS. This increase in NeuN-positive cells suggests improved neuronal survival and preservation within critical hippocampal regions, which are particularly vulnerable to TBI-induced damage. The hippocampus is essential for cognitive and memory functions and preserving neuronal integrity in this area is likely crucial for achieving sustained neurological improvements. Consistent with these tissue-level changes, paracrine signaling has been proposed as a key contributor to early benefits.^42^

Immunofluorescence analyses indicated that HMNS transplantation mitigated multiple TBI-related pathologies—including neuronal degeneration (Fluoro-Jade B), nitrosative/oxidative stress (nitrotyrosine, 4-HNE), and dendritic loss (MAP2). At 7 days post-injury, TBI markedly increased FJB-positive neurons in the ipsilateral hippocampus and elevated nitrotyrosine and 4-HNE immunoreactivity; HMNS treatment significantly reduced these readouts, indicating attenuation of secondary injury.^43,44^ This antioxidative pattern is consistent with prior evidence that MSCs/MSC-derived secretomes modulate the local redox milieu and protect neighboring neurons.^42^ Furthermore, preservation of MAP2 signal in the CA1 and dentate gyrus suggests maintenance of dendritic architecture essential for synaptic connectivity.^45–47^ Collectively, these data support that HMNS provide acute neuroprotection by limiting cellular degeneration and maintaining structural integrity after TBI.

Several limitations warrant consideration. First, we did not include a direct benchmark against conventional MSC therapy—e.g., a cohort receiving non-encapsulated, dissociated hUC-MSCs—or a “neurospheres without hydrogel” group, which prevents quantification of the incremental benefit of our platform. Second, the 7-day observation window precludes conclusions about sustained or long-term functional recovery. Third, in the absence of *in vivo* cell-tracking, we cannot confirm migration of transplanted cells to the lesion.

However, it is well-established that intranasally administered agents can bypass the blood–brain barrier and reach the brain directly via olfactory and trigeminal neural pathways,^48–50^ supporting the biological plausibility of this delivery route. While our data strongly suggest that paracrine mechanisms mediate the observed therapeutic benefits, the ultimate fate of the transplanted hUC-MSCs remains to be defined. Therefore, future studies in our group will focus on performing immunohistochemistry for human-specific markers (e.g., HuNu) to assess the long-term survival, engraftment, and potential differentiation of the transplanted cells within the host brain.

Furthermore, the current study's conclusions could be strengthened by the inclusion of additional control groups to benchmark the therapeutic effects. A direct comparison against a group treated with an equivalent number of dissociated hUC-MSCs encapsulated in the same hydrogel would be necessary to confirm the added value of the neurosphere configuration. Similarly, a group receiving neurospheres without the hydrogel would help isolate the specific contribution of the hydrogel encapsulation to cell survival and retention. We acknowledge that the absence of these controls is a limitation, and these experiments are a key priority for our future work to dissect the relative contributions of each component of our therapeutic system. We also recognize that the *in vitro* mechanical scratch assay is a simplified model that does not recapitulate the complex biochemical environment of *in vivo* TBI, which includes critical factors, such as inflammation, ischemia, and shear stress. Thus, the *in vitro* findings should be interpreted as a reductionist complement to our *in vivo* data. Finally, we did not conduct single-cell transcriptomics, lineage tracing/electrophysiology, or comprehensive immunophenotyping (e.g., SOX2, Nestin, Musashi-1, PAX6, βIII-tubulin, and GFAP) of the neurospheres used in this study. As a result, the precise neural lineage composition and differentiation state of the transplanted HMNS remain undefined, and we do not assign a definitive neural stem/progenitor cell identity in this work, although similar hUC-MSC-derived neurospheres have been phenotypically characterized in prior studies.^34–36^ Future studies will extend beyond the scope of this proof-of-concept work by performing systematic immunocytochemistry and/or qPCR for neural lineage markers on the same HMNS product used for transplantation, in parallel with functional assays.

Despite these limitations, our findings provide compelling preclinical evidence supporting trans-septal delivery of hydrogel-encapsulated HMNS as a biologically plausible and functionally effective therapeutic strategy for TBI.

## CONCLUSIONS

This study demonstrates the significant therapeutic potential of human umbilical cord mesenchymal stem cell (hUC-MSC)-derived neurospheres, encapsulated within a silk fibroin/GelGMA-based hydrogel (HMNS), delivered through the trans-septal administration for the treatment of traumatic brain injury (TBI). *In vitro*, HMNS enhanced cell viability and migration, while *in vivo* transplantation in a rodent TBI model led to improved neurological function, restoration of hippocampal BDNF expression, preservation of neuronal populations, reduction of oxidative and nitrosative stress, and maintenance of dendritic integrity. These findings suggest that HMNS effectively modulates the post-injury microenvironment, exerts acute neuroprotective effects and leads to early improvements in neurological function. In conclusion, hydrogel-encapsulated HMNS represent a promising regenerative therapeutic strategy for TBI. Future research should focus on optimizing delivery protocols, elucidating underlying mechanisms of action, and advancing clinical translation to improve patient outcomes.

## METHODS

### Isolation of human umbilical cord mesenchymal stem cells (hUC-MSC)

Cells were obtained from HansBiomed Co., Ltd. (Seoul, Korea) and cultured according to the manufacturer's instructions. Human umbilical cord (hUC) tissues were disinfected with 1% penicillin/streptomycin in Dulbecco's Phosphate Buffered Saline (DPBS). The sanitized tissues were then minced into approximately 0.5 cm pieces using a pair of sterile scissors. The tissue fragments were incubated with a mixture of DPBS and Trypsin at 37 °C for 30 min to dissociate the cells. After neutralizing trypsin, the cell suspension was transferred to a T75 flask and cultured in minimum essential medium α (MEM α) supplemented with 10% fetal bovine serum (FBS) and 1% penicillin/streptomycin. The culture flasks were maintained at 37 °C in a humidified atmosphere containing 5% CO_2_. Within a week, fibroblast-like cells began to proliferate from tissue fragments. Once the cells reached approximately 80% confluence, they were subcultured using TrypLE™ to initiate passage 1.

### Manufacturing a master cell bank (MCB) through expansion of hUC-MSC in the bioreactor

The quantum cell expansion set was initially conditioned with DPBS and subsequently coated with fibronectin (5 mg/ml in DPBS) for 24 h. After thorough rinsing with DPBS, the system was filled with minimum essential medium α (MEMα) enriched with 10% FBS and 1% penicillin/streptomycin. A controlled gas environment comprising 21% O_2_, 5% CO_2_, and 74% N_2_ was maintained within the system. A suspension of MSCs was prepared by diluting the cells in the growth medium to a final volume of 100 ml, which was then transferred to the Cell Inlet Bag. These cells were seeded into the hollow fibers at a density of 3000 cells/cm^2^ and allowed to adhere for 24 h.

After the attachment phase, a continuous flow of the medium was initiated through the inlet, starting at a flow rate of 0.1 l/min. The flow rate was dynamically adjusted based on the real-time monitoring of lactate and glucose concentrations. If lactate levels stabilized and glucose levels significantly decreased, the flow rate was increased to ensure an adequate supply of glucose. The complete medium was continuously supplied, gradually increasing to a flow rate of 2.0 ml/min while maintaining lactate levels within the range of 4–5 mM. Cell growth was assessed by analyzing glucose consumption and lactate production rates. Cells were harvested after approximately 7 days when the estimated cell count reached 8.00 × 10^8^ to 1.00 × 10^9^ cells. The harvested cells (1.00 × 10^9^ cells) were cryopreserved at passage 3 (P3) at a concentration of 2.00 ×10^6^ cells/vial to establish a master cell bank (MCB) comprising 500 vials.

### Flow cytometry

Mesenchymal stem cells (MSCs) stored in the MCB were phenotypically characterized using flow cytometry on a FACS Verse system (BD Biosciences, USA). To assess their surface marker expression, cells were analyzed for the presence of positive markers (CD44, CD73, CD90, and CD105) and the absence of negative markers (CD14, CD19, CD34, and CD45) according to the International Society for Cellular Therapy (ISCT) criteria defined by Dominici *et al.* in 2006^51^ For flow cytometry analysis, cells were suspended in FACS buffer (99% DPBS, 1% FBS) at a concentration of 2.0–3.0 × 10^6^ cells/ml. A 100 *μ*l volume of the cell suspension was added to each tube, where it was incubated with either isotype control or specific antibodies targeting the positive and negative markers for 20 min at room temperature in the dark. After incubation, 200 *μ*l of FACS buffer was added, and the samples were centrifuged at 300 × g for 5 min. The supernatant was discarded, and the cell pellet was resuspended in 500 *μ*l of FACS buffer and then transferred to 5 ml tubes. Flow cytometry data acquisition was performed on a Verse flow cytometer using FACSuite software (BD Biosciences, USA). The results confirmed that the MSCs expressed the characteristic surface markers CD44, CD73, CD90, and CD105, while lacking the hematopoietic markers CD14, CD19, CD34, and CD45.

### Differentiation of hUC-MSCs

To induce differentiation into adipocytes, osteoblasts, and chondrocytes, mesenchymal stem cells (MSCs) were seeded into six-well plates at a density of 1.0 × 10^5^ cells per well. Cell confluence was monitored over a 24 to 72-h period after seeding, and the culture medium was replaced every three days for 2 weeks using adipogenesis and osteogenesis differentiation kits (Promocell, Heidelberg, Germany). For chondrogenesis, MSCs were cultured at a density of 2.0 × 10^5^ cells per well in chondrogenic induction medium (Promocell, Germany) for 3 weeks.

To confirm successful differentiation, cells were subjected to various staining techniques. Adipocyte differentiation was verified using Oil Red O staining (Sigma Aldrich), osteoblast differentiation was confirmed with alizarin red S staining (Sigma Aldrich), and chondrocyte differentiation was assessed using alcian blue staining (Sigma Aldrich).

### Preparation of silk fibroin (SF)

Silk cocoons were initially disintegrated and degreased by boiling them in a 0.02 M sodium carbonate (Na_2_CO_3_) solution at 95 °C for 40 min. This process effectively removed lipids and extracted sericin proteins. After boiling, the silk fibers were thoroughly washed with distilled water and dried at 60 °C to remove residual chemicals. The dried silk fibers were then dissolved in a solvent mixture composed of water, ethanol, and calcium chloride (CaCl_2_) at a molar ratio of 8:2:1. This dissolution process was carried out at 98 °C for 1 h to obtain a silk protein solution. After a 3-day incubation period, the solution was filtered through a microfilter (Calbiochem, San Diego, CA, USA) to eliminate impurities. The filtered silk protein solution was then freeze-dried to produce a dry silk protein powder with an approximate concentration of 5% (w/v). The purified silk protein powder was stored at 4 °C for future use.

### Preparation of GelGMA

Porcine skin-derived gelatin (Sigma Aldrich, 5 g) was dissolved in 250 ml of double-distilled water at 60 °C for 3–4 h, with constant stirring. The pH of the gelatin solution was adjusted to 3.5 using 1M hydrochloric acid (HCl). Glycidyl methacrylate (GMA, Sigma Aldrich, 10 ml) was gradually added to the gelatin solution at a rate of 0.5 ml/min using a 10 ml syringe, while the mixture was maintained at 75 °C in an oil bath. This reaction proceeded for 24 h with continuous stirring to ensure complete cross-linking between gelatin and GMA. The resulting GelGMA solution was filtered through a microfilter to remove any insoluble particles. Next, the solution was dialyzed against distilled water for 3 days using a dialysis membrane with a molecular weight cutoff of 12 400 Da. The dialysis water was replaced every 12 h to efficiently remove unreacted chemicals. Finally, the dialyzed GelGMA solution was cast into a square plate, frozen at −80 °C, and lyophilized to obtain a dry GelGMA powder.

### Preparation of GelGMA + SF hydrogels

A low-glucose Dulbecco's Modified Eagle Medium (DMEM) solution was prepared by adding 0.3% (w/v) of the photoinitiator lithium phenyl-2,4,6-trimethylbenzoylphosphinate (LAP; Sigma Aldrich) and followed by filtration through a 0.2 μm syringe filter. Lyophilized GelGMA was then dissolved in this DMEM solution to achieve a concentration of 30% (w/v). The solution was incubated at 60 °C to ensure complete dissolution of the GelGMA. SF was dissolved in the DMEM solution at concentrations of 6%. The SF solution was homogenized using a planetary vacuum mixer (ARV-310, Thinky Corp., Tokyo, Japan) and subsequently filtered through a 40 μm cell strainer (SPL Co., Pocheon, Gyeonggi, Korea). The homogenized SF solution was sterilized at 60 °C for 60 min. Finally, the 30% GelGMA solution and the 6% SF solution were combined in a 1:1 ratio, resulting in a hydrogel that contained 15% GelGMA and 3% SF.

The hydrogel consisted of 15% (w/v) GelGMA and 3% (w/v) silk fibroin. This composition was selected based on our prior optimization studies,^33^ as it provides brain-compatible mechanical stiffness (∼1–2 kPa) and a degradation rate that supports cell viability and local retention after transplantation. Immediately before administration, partial pre-polymerization of the hydrogel precursor was performed under UV light. This crucial step increases the viscosity of the solution to minimize dispersion during the trans-septal injection and to ensure controlled *in situ* gelation within the targeted mucosal pocket.

### Generation of neurospheres from hUC-MSCs

Human umbilical cord-derived mesenchymal stem cells (hUC-MSCs) were cultured in Minimum Essential Medium Alpha (MEM α, Gibco, 12571-063) supplemented with 10% fetal bovine serum (FBS) and 1% penicillin-streptomycin (p/s). Cells at passages 3–6 were harvested and used for neurosphere formation. To induce neurosphere formation, 5.00 × 10^6^ to 1.00 × 10^7^ cells were seeded in 100 mm ultra-low attachment plates containing neurosphere formation medium. This medium is composed of DMEM/F-12 supplemented with 2% B27, 20 ng/ml basic fibroblast growth factor (bFGF), 20 ng/ml human epidermal growth factor (hEGF), and 0.2% penicillin-streptomycin. Neurospheres were cultured for 5 days with media changes every 2–3 days. Once neurospheres had formed, they were transferred to neurobasal medium supplemented with 5% FBS, 1% L-glutamine, 1% non-essential amino acids (NEAA), 1% N2 supplement, 2% B27, and 0.2% penicillin-streptomycin to induce neuronal differentiation. The differentiated neurons were subsequently used for *in vitro* or *in vivo* experiments.

### *In vitro* TBI-mimicking scratch assay

Human umbilical cord-derived mesenchymal stem cells (hUC-MSCs) were encapsulated in hydrogels and cultured overnight on various cell culture inserts in DMEM/F-12 supplemented with 2% B27, 2% FBS, 20 ng/ml basic fibroblast growth factor (bFGF), 20 ng/ml human epidermal growth factor (hEGF), and 0.2% penicillin-streptomycin.

HT22 mouse hippocampal neuronal cells were cultured in DMEM supplemented with 10% FBS and 1% penicillin-streptomycin. Cells from passages 3–6 were collected and seeded at a density of 1 × 10^4^ cells/ml in 24-well plates. Following an overnight incubation, a scratch injury was inflicted on the cell monolayer using a sterile 10 *μ*l pipette tip to mimic traumatic brain injury. This scratch injury created a 4 × 4 grid of wounds in each well. The cultures were then incubated without a medium change.

After overnight culture, encapsulated hUC-MSC neurospheres in transwell inserts containing 15% GelGMA and 3% silk fibroin (SF) were added to each well. After a 48-h incubation period, cell viability was assessed using a Cell Counting Kit-8 (CCK-8, Enzo Life Sciences, Farmingdale, NY, USA) assay. For all experiments, the primary control group was the hydrogel-only (vehicle) group, which consisted of the acellular silk-fibroin/GelGMA hydrogel. For the therapeutic group, neurospheres were first generated as described above (pre-formed). These fully formed neurospheres were then gently mixed into the hydrogel precursor solution immediately prior to use for encapsulation. No in-gel differentiation was performed.

### Establishment of a controlled cortical impact (CCI) model of TBI

All animal studies were conducted according to the National Institutes of Health guidelines under the approval of the Committee on Animal Use for Research and Education at Hallym University (protocol # Hallym 2018-83).

Rats were anesthetized using a 3% isoflurane-oxygen mixture delivered via a vaporizer. The animals were then fixed in a stereotaxic apparatus and maintained on 1–1.5% isoflurane during the surgical procedure. A craniotomy was performed to expose the right hemisphere, creating a 5 mm diameter opening 2.8 mm lateral to the midline and 3 mm posterior to the bregma. A controlled cortical impact device was used to inflict a TBI by delivering a 3 mm flat-tip impactor at a velocity of 5 m/s to a depth of 3 mm. To maintain body temperature throughout the procedure and during the recovery period, the animals were placed on a heating pad set to 36–37.5 °C. In this study, behavioral and histological outcomes were assessed at 7 days post-injury to capture the acute neuroprotective effects. A sham-operated group, which underwent only the craniotomy procedure, served as a control.

### Trans-septal cell transplantation in a rat TBI model

Sprague–Dawley male rats (8 weeks) were used for the trans-septal transplantation procedure. Following the induction of TBI in the rats, the nasal area was disinfected using a 10% Povidone-Iodine swab stick. Local anesthesia was administered by injecting 1 ml of 1% lidocaine with epinephrine (1:100 000) along the nasal dorsum before cutting the skin and subcutaneous tissues open. A fracture was created in the right nasal passage using surgical scissors to access the septum. A pocket was then formed between the nasal septum and the mucosa for transplantation of hydrogel-encapsulated neurospheres. Neurospheres (200–400 μm in diameter) were mixed with a hydrogel precursor solution made up of 15% (w/v) GelGMA + 3% (w/v) SF at a concentration of 200 neurospheres per milliliter of the precursor solution under sterile conditions. The hydrogel precursor solution was prepared with a LAP-containing medium as discussed above.

Using a Hamilton syringe with a blunt-tip 21G needle, 100 *μ*l of hydrogel precursor containing about 200 neurospheres was partially polymerized with UV light of 2 mW/cm^2^ intensity before injecting 20 *μ*l of the partially polymerized neurospheres containing hydrogel into the pocket within 10 min post-TBI. Control groups received an equal volume of the acellular silk-fibroin/GelGMA hydrogel, hereafter referred to as the vehicle (hydrogel-only). All groups were monitored for 7 days after surgery to assess neurological outcomes.

### Tissue preparation

Rats were deeply anesthetized with urethane administered intraperitoneally at a dose of 1.5 g/kg in saline. Once anesthesia was confirmed, the animals underwent cardiac perfusion with saline followed by fixation with 4% paraformaldehyde in phosphate-buffered saline (PBS). The brains and septum were further post-fixed in 4% paraformaldehyde for an hour and then cryoprotected in 30% sucrose solution. After cryoprotection, the entire brain was frozen and sectioned into 30 μm-thick coronal slices using a cryostat microtome.

### Assessment of neurological deficits

To assess the efficacy of hUC-MSC-laden hydrogels in mitigating neurological deficits caused by TBI, we employed the modified neurological severity score (mNSS) to quantify neurological function. The mNSS was administered daily for one-week post-TBI or sham surgery. This test evaluates motor, sensory, balance, and reflex functions, with higher scores indicating greater neurological impairment. All behavioral assessments were performed by two independent evaluators who were blinded to the treatment groups to ensure unbiased scoring. Inter-rater agreement was verified prior to the final data analysis to ensure consistency. The modified neurological severity score (mNSS) is a composite functional test that evaluates tail flick response, gait, sensory function, balance, and motor coordination, with each item scored on a scale of 0–3. The total mNSS, obtained by summing these subscores, provided a quantitative measure of neurological injury severity.^52^

### Assessment of neuronal death

To assess neuronal death following TBI, Fluoro-Jade B (FJB) staining was performed 7 days post-injury. Six coronal brain sections, spaced 270 *μ*m apart, were collected from each animal, spanning the region from 2.92 to 4.56 mm caudal to bregma. These sections were examined under a confocal microscope. A blinded observer quantified neuronal damage in the ipsilateral hemisphere by counting FJB-positive neurons in the CA1 region of the hippocampus, the granule cell layer (GCL), and the hilus. The average number of FJB-positive neurons in each region was used to represent the extent of neuronal loss.^43^

### Immunofluorescence analysis

Immunofluorescence labeling was carried out using established immunostaining protocols. Sections were initially treated with 1.2% hydrogen peroxide for 20 min at room temperature to block endogenous peroxidase activity. After washing, the sections were incubated overnight at 4 °C with a mix of polyclonal and monoclonal primary antibodies in PBS containing 0.3% Triton X-100. The antibodies used in this study included MAP2 (1:500, Abcam), 4-HNE (1:500, Alpha Diagnostic International), nitrotyrosine (1:500, Abcam), BDNF (1:400, Alomone Labs), and NeuN (1:500, Millipore). For double labeling, the antibodies were applied together. After a washing step in PBS, sections were incubated with secondary antibodies conjugated to fluorescent dyes (1:250, Invitrogen), followed by counterstaining with DAPI (1:1000, Invitrogen). The sections were mounted on gelatin-coated slides and coverslipped with DPX (Sigma-Aldrich). Fluorescence images were captured using a confocal microscope (LSM 710; Carl Zeiss).

### Statistical analysis

All statistical analyses were performed using GraphPad Prism (GraphPad Software, San Diego, CA). Data are presented as mean ± standard error of the mean (SEM). For comparisons between two groups, an unpaired, two-tailed Student's t-test was used. For comparisons involving three or more groups, a one-way analysis of variance (ANOVA) was performed, followed by Tukey's post hoc test for multiple comparisons. A p-value of less than 0.05 was considered statistically significant. Specific statistical details, including exact n values for each group, are provided in the respective figure legends.

## Data Availability

The data that support the findings of this study are available from the corresponding author upon reasonable request.
